# Allopregnanolone and neurogenesis in the nigrostriatal tract

**DOI:** 10.3389/fncel.2014.00224

**Published:** 2014-08-12

**Authors:** Jun Ming Wang

**Affiliations:** Departments of Pathology, Psychiatry and Human Behavior, and Pharmacology and Toxicology, Memory Impairment and Neurodegenerative Dementia Center, University Mississippi Medical CenterJackson, MS, USA

**Keywords:** allopregnanolone, neurogenesis, substantia nigra, nigrostriatal, tyrosine hydoxylase, neural circuits, motor performance

## Abstract

Reinstalling the neurobiological circuits to effectively change the debilitating course of neurodegenerative diseases is of utmost importance. This reinstallation requires generation of new cells which are able to differentiate into specific types of neurons and modification of the local environment suitable for integration of these new neurons into the neuronal circuits. Allopregnanolone (APα) seems to be involved in both of these processes, and therefore, is a potential neurotrophic agent. Loss of dopamine neurons in the substantia nigra (SN) is one of the main pathological features of Parkinson’s and also in, at least, a subset of Alzheimer’s patients. Therefore, reinstallation of the dopamine neurons in nigrostriatal tract is of unique importance for these neurodegenerative diseases. However, for the neurogenic status and the roles of allopregnanolone in the nigrostriatal tract, the evidence is accumulating and debating. This review summarizes recent studies regarding the neurogenic status in the nigrostriatal tract. Furthermore, special attention is placed on evidence suggesting that reductions in allopregnenalone levels are one of the major pathological features in PD and AD. This evidence has also been confirmed in brains of mice that were lesioned with 1-methyl-4-phenyl-1,2,3,6-tetrahydropyridine (MPTP) or those bearing neurodegenerative mutations. Lastly, we highlight studies showing that allopregnanalone can augment the number of total cells and dopaminergic neurons via peripheral exogenous administration.

## Introduction

Research data consistently suggests that a small molecule, the neurosteroid allopregnanolone (APα) capable of permeating the brain-blood-barrier, is a latent restorative therapeutic agent for reestablishing neuronal circuits in hippocampus and also the nigrostrital tract. Supportive data demonstrated that APα functioned as a neurotrophic factor for human, rat, and mouse neural progenitor cells (Keller et al., 2004; Wang et al., 2005, 2010; Charalampopoulos et al., 2008) and augmented the number of cells in the hippocampus and reversed deficits in learning and memory in a mouse model for Alzheimer’s disease (3xTgAD, a triple transgenic with APPSwe, PS1M146V, tauP301L) (Wang et al., 2010; Chen et al., 2011; Singh et al., 2012), for review see Brinton (2013) and Irwin and Brinton (2014). In contrast, APα has been reported to inhibit the learning and memory when chronically treated for 3 months (Bengtsson et al., 2012, 2013) and the potential mechanisms for this discrepancy have been discussed elsewhere (Brinton, 2013; Wang, 2013; Irwin and Brinton, 2014). In addition, APα also plays a role in regulating depressive episodes (Schüle et al., 2011, 2014; Evans et al., 2012; Hellgren et al., 2014) and the antidepressant effects of APα is probably mediated via neurogenesis in dentate gyrus in hippocampus (Evans et al., 2012). Recently, accumulated data indicated that APα increased the number of total cells, tyrosine hydroxylase (TH) positive cells, and newly formed (BrdU positive) TH expressing cells in the substantia nigra (SN), and improved the balance and coordination of 1-methyl-4-phenyl-1,2,3,6-tetrahydropyridine (MPTP)-lesioned mice, an animal model for Parkinson’s disease (PD; Adeosun et al., 2012). The augmentation of TH positive neurons by APα in the SN of 3xTgAD mice (Sun et al., 2012a) clarified that APα accomplished its role through the reestablishment of DA neuronal architecture, rather than blockading the neurotoxic effects of MPTP. This review summarizes and highlights the current discoveries involving the generation of new neurons in the nigrostriatal tract and the therapeutic potential for the related neuronal disorders of APα.

## The significance for A therapeutic strategy to reinstall the functional DA neurons in nigrostriatal tract

AD and PD are devastating, degenerating neural disorders which currently cannot be cured. More than 5 million and nearly 1 million Americans have AD or PD, respectively, and every minute a new case is added in this cohort. These diseases not only bring about suffering for the patients themselves, it is also a heavy financial burden and high labor cost for both the families and society. The severity of these diseases is closely related to the number of neurons that are lost in specific brain regions.

For example, the symptoms of PD are closely associated with the depletion of striatal dopamine (DA), brought on by the impairment of normal neurobiological architecture of neural cells in the nigrostriatal tract, resulting in the degeneration and death of DA neurons. The role of nigrastriatal tract in AD has been reported in studies using post-mortem brains from patient with AD, transgenic mice with human AD mutations, and also from those studying the dopamine effects in AD (Uchihara et al., 1992; Love et al., 1996; Perez et al., 2005; Nardone et al., 2014). Diffuse plaques in the striatum and neurofibrillary tangles in the SN were consistent findings in all of the Alzheimer brains tested (Uchihara et al., 1992; Love et al., 1996). Furfuremore, a 41% significant neuronal loss was observed in SN of AD subjects compared to that in the age matched controls (Uchihara et al., 1992). Although in the study by Love et al. (1996), quantitation did not reveal a statistically significant correlation between the density of striatal plaques and the numbers of either neurofibrillary tangles or neurons in the SN in “pure” AD (i.e., without clinical or neuropathological evidence of Parkinson’s or cortical Lewy body disease), the mean number of neurons in the SN of Alzheimer brains was lower than that in controls (Love et al., 1996). Pharmacologically, L-Dopa significantly increased Short-latency afferent inhibition (SAI) in the AD patients, while it failed to restore SAI abnormality in patients with Cerebral Autosomal Dominant Arteriopathy with Sub-cortical Infarcts (Nardone et al., 2014). Therefore, L-Dopa-mediated changes on SAI in AD patients seem to be a specific effect. The striatum and the SN of transgenic mice harboring familial AD (FAD)-linked APPswe/PS1DeltaE9 mutants exhibit morphological alterations accompanied by amyloid-beta (Aβ) deposition 6 months of age, and the extent of deposition increases in an age-dependent manner (Perez et al., 2005). In addition, a reduction in the dopamine metabolite DOPAC was also observed in the striatum of these mice (Perez et al., 2005). These findings suggested a close association between amyloid deposition and nigrostriatal pathology and suggest that altered familial AD-linked amyloid metabolism impairs, at least in part, the function of dopaminergic neurons.

L-DOPA treatment and deep brain stimulation only provide symptomatic relief by increasing brain DA levels without altering the course of the disease. Scientists have been attempting to adjust the developmental course of the disease by restoring region-specific DA neuron architecture (Soderstrom et al., 2006). Initial trials to replenish DA neurons used grafts of DA-producing adult adrenomedullary tissue and then fetal mesencephalic tissue (Collier et al., 2002; Williams and Lavik, 2009; Lindvall and Kokaia, 2010), but encountered many obstacles (Björklund, 1993). Recent discoveries overcame these hurdles by generating patient-derived pluripotent and growth factor-enhanced fibroblasts to increase the supply of tissue for grafting and to prevent transplant rejection. However, new problems have materialized. It is still not clear whether grafted cells will survive in a pathological environment with a deteriorated milieu and be appropriately integrated into a >50-year-old local neuronal network. In fact, patients in clinical trials with grafted cells emerged with dyskinesia (Freed, 2002; Maries et al., 2006). This data implicates that the grafted new cells were not integrated into the existing neuronal network and were not capable of performing the expected functions. Interestingly, PD pathological markers, Lewy bodies and α-synuclein aggregates, had been observed in the grafted cells (Li et al., 2008; Hansen et al., 2011). Thus, there is an urgent need for a therapeutic strategy to restore functional DA neurons, which could effectively integrate into the existing neuronal network.

## APα improves balance and coordination and increases the number of new tyrosine hydroxylase cells in SNpc of MPTP-lesioned mice

**Figure 1:**
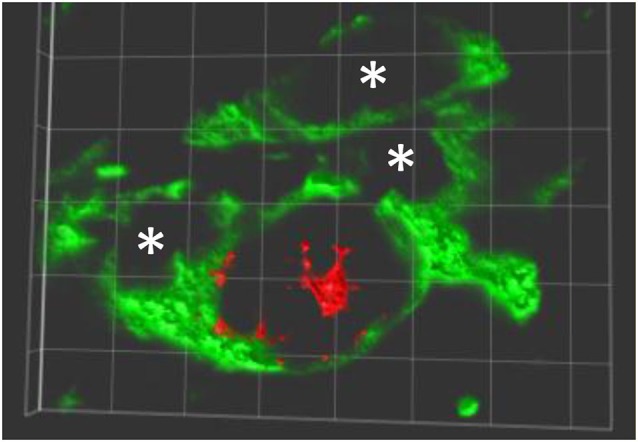
**New TH expressing neurons in SNpc of allopreganolone treated mice lesioned by MPTP**. A 3-dimensionally rotated double-immunolabeling image shows a new neuron (red, BrdU positive in nuclear) expressing TH (green in cytoplasm and neurites), and a few TH positive only neurons marked with *. Similar image can be found in Adeosun et al. (2012).

MPTP-lesion impairs the motor performance, particularly in the modalities of balance and coordination, in C57BL/6J mice (Antzoulatos et al., 2010). The balance and coordination of MPTP-lesioned mice were improved in a rotarod performance task in which mice were forced to move correctly to prevent them from falling. Mice that received peripheral administration of APα almost completely regained their ability to walk on the rod (Adeosun et al., 2012). Correlated to the improvement of motor performance, the number of tyrosine hydroxylase immunoreactive (TH-IR) cells in the SN in APα-treated, MPTP-lesioned mice was increased. This data suggests that APα promotes the reinstallation of functional neural circuits in the nigrostriatal pathway either by reversal (recovery) of the MPTP-induced degeneration of TH neurons, and/or the generation of new (or differentiated) TH-expressing neurons in this brain region. In addition to the increase of TH-IR neurons, APα also increased the number of Nissl stained cells, which were both reduced in mice only received the MPTP neurotoxin. These results, in addition to the increase of BrdU/TH double positive cells in APα-treated mice, suggest that new cells, not only TH-IR neurons but also the non-neuronal cells, were added into the SN of the MPTP-lesioned mice (Adeosun et al., 2012; Figure 1).

It is still in debate whether neurogenesis also occurs in the SN. Studies from different groups demonstrated that new cells were born in the healthy SN (Lie et al., 2002; Zhao et al., 2003). The precursor cells isolated from the SN had the ability to differentiate into neurons *in vitro* (Lie et al., 2002) or the generation of new mature nigral DA neurons under physiological conditions by colocalization of BrdU and TH (Zhao et al., 2003). In contrast, opposite report indicated that there is no evidence for neurogenesis in SN (Frielingsdorf et al., 2004) and argued that the BrdU and TH co-localization was an overlay of a BrdU positive glia on an adjacent neuron (Borta and Hoglinger, 2007). However, a number of works have also described the expression of polysialylated-neural cell adhesion molecule (PSA-NCAM), a molecular expressed in multipotent progenitor cells, in the cells of SN (Nomura et al., 2000; Yoshimi et al., 2005) and a small number of cells are PSA-NCAM double positive (Yoshimi et al., 2005). Borta and Hoglinger (2007) discussed that PSA-NCAM is also expressed in other cells undergoing plastic changes, and therefore, these results do not support the hypothesis of dopaminergic neurogenesis in the SN. Peng et al. reported that fibroblast growth factor 2 increased the number of BrdU and doublecortin double positive cells in SN of MPTP-lesioned mice (Peng et al., 2008). Others reported that either physical activity or Unilateral lesion of the subthalamic nucleus increased the oligodendrogenesis and astrogliogenesis in the SN after 6-OHDA lesion (Steiner et al., 2008; Klaissle et al., 2012). Recently, it was also reported that the majority of newly generated cells in the adult mouse SN express low levels of doublecortin (Worlitzer et al., 2013). Taken together, these data support the generation of new cells in SN, but whether these new cells will differentiate into functional DA neurons is not clear. Perhaps by reestablishing the extracellular milieu and local environment in SN to a level suitable for new neuron differentiation, maturation and integration into the existing neuronal circuits will be a hopeful solution. In addition, appropriate labeling protocols may be needed to identify the newly generated neurons by optimized amount of BrdU (Zhao and Janson Lang, 2009), or by tracing the ratio of C^14^ in DNA of cells in striatum of human brains (Ernst et al., 2014).

Accumulated evidence suggests that there are multiple neurogenic niches in the brain apart from the hippocampal dentate gyrus sub granular zone (SGZ) and the cerebral sub ventricular zone (SVZ). These include the hypothalamus (Lee et al., 2012), cerebellum (Keller et al., 2004; Ponti et al., 2005, 2006, 2008, 2010; Bonfanti and Ponti, 2008; Hajihosseini et al., 2008), striatum (Tattersfield et al., 2004; Ninomiya et al., 2006; Luzzati et al., 2007; Snyder et al., 2010; Danilov et al., 2012; Delavaran et al., 2013; Ernst et al., 2014; Kempermann, 2014), and SN (Bayer et al., 1995; Zhao et al., 2003; Chen et al., 2005; Van Kampen and Robertson, 2005; Yoshimi et al., 2005; Arias-Carrión et al., 2006, 2009; Freundlieb et al., 2006; Shan et al., 2006; Steiner et al., 2006; Esposito et al., 2007; Mandel et al., 2007; Di Giovanni et al., 2009; Ries et al., 2009; Park et al., 2012; Sun et al., 2012a,b; Worlitzer et al., 2013). Therefore, APα may promote the generation of new cells locally in SN. One such possibility is that APα increases proliferation of glial fibrillary acidic protein (GFAP), or Ng2 expressing glia cells, which maintain their mitotic status, and drives the differentiation of these new cells into DA neurons in the SN. This hypothesis is supported by the recent studies demonstrating that the primary progenitors in adult neurogenesis are astrocyte-like cells that express GFAP and that surviving cells exhibit neurites 7 days after proliferation (Cabras et al., 2010; Ming and Song, 2011). Furthermore, it has also been reported that, in the presence of sonic hedgehog, GFAP-expressing mesencephalic progenitor cells can be differentiated into TH-IR neurons within 4 days (Matsuura et al., 2001). Parallelly, the new adult subependyma cells (BrdU positive) of the lateral ventricle can differentiate into TH-expressing neurons after 24-h exposure to fibroblast growth factor (bFGF2) and glial cell conditioned media (Daadi and Weiss, 1999). Therefore, it is possible that in the SN, the proliferating glial-like cells have the capacity to differentiate into both neurons and glial cells as regulated by their microenvironment. This is further supported by a recent study which demonstrate that glia cells can differentiate into neurons in the presence of neuronal differentiation 1, a basic helix-loop-helix transcription factor (Guo et al., 2014).

## APα is a potential neurogenic agent in nigrostriatal tract

The neurotrophic feature of APα is widely supported by the literature. APα is produced in pluripotent progenitor cells (Lauber and Lichtensteiger, 1996; Gago et al., 2004) and neurons (Pinna et al., 2004; Agís-Balboa et al., 2006) throughout the embryonic period. In late gestation, a developing period in which large amount of CNS neurons are generated and functional circuits are formed, APα concentration is 20–30 times higher than any other time in life (Pomata et al., 2000). In pathological conditions, the concentration of APα is significantly reduced in the brains of humans with AD (Marx et al., 2006; Smith et al., 2006; Naylor et al., 2010), with PD (di Michele et al., 2003; Luchetti et al., 2010) as well as from the brains of a transgenic mouse model of AD (Wang et al., 2007, 2010). More interestingly, the lower the APα concentration, the more severe these neurodegenerative diseases are and the pathology appears to be inversely correlated with the levels of APα (Naylor et al., 2010).

In mice lesioned with MPTP, APα reversed the cell number decline of TH-expressing and Nissl positive cells in both SN and Locus coeruleus (LC; Adeosun et al., 2012). This data suggest that the generation of new cells by APα is not cell type, brain region, or mouse model specific, as we previously reported the neurogenic property of APα in the SGZ and SVZ in a mouse model of AD (Wang et al., 2010; Chen et al., 2011; Singh et al., 2012). The fact that APα increased the proliferation of cerebellar neurogenic cells supports the observation that APα is not only a neurogenic agent in known neurogenic areas such as SGZ and SVZ, but also in brain regions such as the cerebellum (Keller et al., 2004) and the SNpc (Adeosun et al., 2012; Sun et al., 2012a).

Interestingly, it appears that the neurogenic effects of APα need to be enhanced or maintained with physical activities (Adeosun et al., 2012). In support, utilizing running wheels or forced treadmill for several weeks increased the TH expression (Gerecke et al., 2010; Tajiri et al., 2010). Moreover, a significant increase in numbers of newborn NG2-positive and GFAP-positive cells was observed in the SN of 6-OHDA lesioned animals living in enriched environment with physical activity for 7 weeks. These mice showed improved motor behavior compared to controls under standard conditions (Steiner et al., 2006). Therefore, it is likely that forced physical activity helps the survival and differentiation of newly formed cells induced by APα. This point of view is supported by the fact that newly formed neural progenitors can differentiate into TH-expressing neurons within 24 h when exposed to glial cell conditioned media or basic bFGF2 (Daadi and Weiss, 1999), and in line with report that bFGF2 expression is increased after physical exercise (Gómez-Pinilla et al., 1997).

## APα functions in the nigrostriatal tract of mice with AD mutations

Is APα only a blockade for MPTP-lesion, or a neurogenic agent in the SN? Recent work demonstrating that APα also increases TH positive neurons and total cell numbers in the SN of a triple transgenic mouse for AD (3xTgAD) Sun et al. (2012a) clarified that APα played its role through reestablishment of DA neuronal architecture, rather than by the blockade of the neurotoxic function of MPTP. Further support is from reports that genetic risk factors found in familial AD (i.e., mutations in APP, PS1 and tau phosphorylation genes) also play a role in SNpc neuropathology and atrophy.

Besides the occurrence of plaques, tangles and hippocampal atrophy, atrophy in brain nuclei containing TH expressing neurons is also a neuropathological feature of late-onset AD (Chui et al., 1986; LaFerla et al., 1997; Zarow et al., 2003). For example, a meta-analysis concluded a consistently high TH neuron loss (52–76%) in LC, and a variable neuron loss (4–50%) in the SNpc in post-mortem brains of late onset AD subjects (Zarow et al., 2003). These data indicate that reduction of TH expressing and total neurons in SN of animals bearing AD mutations (Sun et al., 2012a) occurs, and is in agreement with those early studies from AD subjects (Zarow et al., 2003).

Supportive evidence was also emerged from the transgenic APP/PS1 mouse model of AD, in which hyper-accumulated Aβ-42 residues lead to the early appearance of amyloid plaque formation when compared to mice with only the single transgene APP (Perez et al., 2005; O’Neil et al., 2007). In the APP/PS1 double mutant mice there was a significant (24%) reduction in TH-positive neurons in the LC in comparison to their background controls (O’Neil et al., 2007). Interestingly, the loss of TH expressing neurons was not observed in the transgenic mouse model with APP23 (Szot et al., 2009) nor PADPP (German et al., 2005). These findings suggest that the loss of TH positive neurons may be a result of the double APP/PS1 mutations, rather than a single APP mutation.

It has been proposed that tau protein abnormalities play a more important role in the loss of neurons in AD, and that deposition of amyloid plaques does not correlate well with neuron loss (Mudher and Lovestone, 2002; Mudher et al., 2004; Schmitz et al., 2004). Neurofibrillary tangle formation is composed of hyperphosphorylated microtubule-associated protein tau that appears to accumulate within vulnerable neurons and may eventually kill the cell, leaving behind only a ghost tangle and no neuron (Ramsden et al., 2005; Iqbal and Grundke-Iqbal, 2006; Gong and Iqbal, 2008). The 3xTg mice carry, in addition to two mutations in human familial AD genes (APP_Swe_, PS1_M146V_), one frontal temporal dementia-linked tau mutation (tau_P301L_) and mimic multiple aspects of AD neuropathology in relevant brain regions (Oddo et al., 2003a,b). The reduction of TH-immunoreactive neurons in the SNpc of 3xTgAD male mice at 3 months old, extend the previous report and supports the hypothesis that early neurogenic deficits lead to the reduction of total neuron numbers in multiple brain regions of AD subjects (Wang and Sun, 2010) including SNpc. SN lesions are frequently present in AD and include pigmented neuronal cell loss, gliosis, Lewy bodies, α-synuclein-stained structures, and hyperphosphorylated tau accumulation in neurofibrillary tangles as well as neuritis (Kazee et al., 1995; Klunk et al., 2004; Burns et al., 2005), suggesting that AD is a significant risk factor for SN lesions (Kazee et al., 1995; Kazee and Han, 1995). APα-induced neurogenesis is dose-dependent and the most effective dose *in vitro* also has neurogenic effects *in vivo* that are accompanied with a reversal of the cognitive deficits in 3xTgAD mice (Wang et al., 2005, 2010). Previous studies indicated biphasic dose-dependent efficacy of APα on neurogenesis (Wang et al., 2005, 2010). At 100, 250, and 500 nM concentrations, APα significantly increased BrdU incorporation (lower concentrations were not statistically different from the control). At 1000 nM, a reversal of the dose–response was first apparent, with higher doses shifting the response to significant repression of proliferation at 100–1000 nM. A recent study titrated the optimal regimen for therapeutic efficacy of APα treatment *in vivo* in 3xTgAd mice (Chen et al., 2011). In both APα treatment regimens of a single exposure of 1/month and of repeated expose (1/week/6 months), APα treatment significantly increased the survival of cells that were generated at the first exposure to APα. The repeated exposure (1/week/6 months) APα treatment regimen had greater regenerative efficacy. However, the 3/week/3 months regimen significantly reduced regenerative efficacy (Chen et al., 2011). This once per week regimen suggested there might be a 7-day cycle which could help reach the best effects of APα and this seems consistent with the role of APα in SN (Adeosun et al., 2012; Sun et al., 2012a).

In contrast, recent work by Bengtsson and colleagues demonstrated that constant infusion of APα (via ALZET mini-pumps) for 3 months increase GABAergic function/inhibition in brain. These levels of APα showed a negative impact on both learning and memory and neuropathology of amyloid beta deposition (Bengtsson et al., 2012, 2013; Wang, 2013). Results of these investigations are in line with learning and memory deficits experienced by people who are chronically treated with high levels of anti-seizure medications. Mechanistically, this may be due to the accumulation of APα in the brain with a final concentration high enough to enter the second phase (inhibition) of the dose response of APα on neurogenesis (Wang et al., 2005; Brinton, 2013; Irwin and Brinton, 2014).

Wang and Brinton reported that APα transiently increases intracellular calcium concentration in primary cultured hippocampal neurons. This intracellular calcium increase is mediated by GABA_A_ receptor and L-type Calcium Channel (Wang and Brinton, 2008) and this calcium increase is related to neural progenitor cell proliferation *in vitro* (Wang et al., 2005) and *in vivo* (Wang et al., 2010). The APα induced transient increase of intracellular calcium concentration and the subsequent proliferation of progenitor cells was abolished by inhibitors for GABA_A_ receptor and voltage gated calcium channel blockers (Wang and Brinton, 2008). Therefore, it is likely the effects of APα on the increase of new neurons and cells are also mediated by GABA_A_ receptor regulated transient increase of calcium concentration, however, more experimental evidence is needed.

## Summary and potential expectation

In summary, research demonstrates that the levels of APα, are reduced in the brains of subjects with AD or PD. The promising role of this APα therapy in AD and PD is supported by the recent work that peripheral administration of APα, with its ability to permeate the blood brain barrier, could improve cognitive and motor performance and increase the number of DA neurons in the SN of mice lesioned by MPTP and mice with AD mutations. These results support that APα accomplishes its role through the reestablishment of dopamine neuronal architecture, rather than blockading the neurotoxic effects of MPTP.

## Conflict of interest statement

The author declares that the research was conducted in the absence of any commercial or financial relationships that could be construed as a potential conflict of interest.
